# Multi-Channel Neighbor Interactions Adjust Growth Strategies in the Clonal Plant *Glechoma grandis*

**DOI:** 10.3390/plants15193060

**Published:** 2026-10-07

**Authors:** Yuzhuo Liu, Yidan He, Jiali Xue, Jing Tang, Shuqi Zhang, Jiaxin Quan, Yue Yang, Ming Yue, Xiao Liu

**Affiliations:** 1 Key Laboratory of Resource Biology and Biotechnology in Western China, Ministry of Education, Northwest University, Xi’an 710069, China; liuyuzhuo2001@163.com (Y.L.); 18634804980@163.com (Y.H.); 19556708608@163.com (J.X.); 16635475021@163.com (J.T.); zhangshuqi137@163.com (S.Z.); yangyue1015@126.com (Y.Y.); yueming@nwu.edu.cn (M.Y.); 2 Xi’an Botanical Garden of Shaanxi Province/Institute of Botany of Shaanxi Province, Xi’an 710061, China; jiaxinquan007@163.com

**Keywords:** plant–plant interactions, leaf touching, shared rooting space, clonal plants, thigmomorphogenesis

## Abstract

**Background:** Plant–plant interactions rely on neighbor-derived cues. Access to multiple cue channels should allow better identity assessment, yet integrative studies—especially on touch between neighbors—remain limited. **Methods:** We tested how leaf contact and shared rooting space between neighbors influence plant–plant interactions, by measuring growth of *Glechoma grandis* paired with conspecific, congeneric, or distant heterospecific neighbors. **Results:** Neighbor effect strength depended on interaction mode: leaf contact alone triggered stronger neighbor identity-dependent growth than shared rooting space alone, and combined channels were strongest. With both channels, plants were a conservative strategy toward conspecifics (reducing stolon allocation), but competitive toward heterospecifics (increasing leaf area and roots, stolons and leaf biomass). The competitive strategy further diverged with phylogenetic distance—congeneric neighbors induced thicker stolons; distant heterospecific neighbors led to longer, thinner stolons. Moreover, interaction mode outweighed neighbor identity: without interactions, neighbor effects differed only between conspecific and heterospecific neighbors (conspecifics induced longer stolons and more ramets, indicating greater expansion, but heterospecific neighbors did not differ); with interactions, leaf contact mainly affected leaf area, shared rooting space affected root allocation, and combined channels eliminated differences in *G. grandis* among neighbors combinations. **Conclusions:** Leaf contact triggered the most obvious neighbor identity-dependent growth responses, whereas the combination of leaf contact and shared rooting space caused convergence of growth responses among neighbor combinations.

## 1. Introduction

Plant–plant interactions, central to community dynamics, are driven by the perception of neighbor-derived signals. These signals range from belowground cues—including root exudates [1,2,3], rhizosphere microbes [4], and electrical signals [5]—to aboveground cues such as shifts in light quality [6], volatile organic compounds [7,8,9,10], and mechanical touch [6,7]. Perception of these signals initiates cascades of transcriptional, metabolic, and morphological responses that ultimately govern interaction outcomes [11].

Among the signals mediating plant–plant interactions, mechanical touch between neighbors—ubiquitous in dense stands—remains understudied. Leaf-tip contact provides the most immediate neighbor detection, preceding even light cues [6,9,12]. This mechanical stimulation triggers morphogenic responses—including upward leaf movement and shifts in growth, development, and defense—that reshape competitive dynamics [13,14,15]. Thus, touch is a key sensory mechanism, yet the ecological consequences of such thigmomorphogenesis are poorly understood. Below ground, root-derived chemical signals mediate self/non-self-recognition [16] and, through modulated root exudate profiles, directly influence neighbor performance, steering interactions toward facilitation or competition [3]. Despite growing knowledge of above- and belowground signaling, studies that integrate both pathways remain limited. A comprehensive approach examining both aerial and subterranean communication is needed to uncover the mechanisms driving plant–plant interactions and to illuminate species coexistence, community dynamics and their evolutionary drivers.

From an evolutionary perspective, selection favors mechanisms that reduce competition among close relatives. Studies of intraspecific plant interactions show that plants typically show reduced competition with kin and intensified it with non-kin [17]. This kin recognition should be particularly pronounced in clonal species, where genetically identical ramets gain strong adaptive benefits from avoiding intra-clonal competition [18]. Evidence from diverse clonal species supports this prediction: in *Ambrosia dumosa*, root growth is inhibited by non-self ramets but not by ramets of the same genet [19]; in *Fragaria chiloensis*, connected ramets exhibit root avoidance [20]; and in *Carpobrotus edulis*, close kin display cooperative behaviors [21]. However, some clonal species, such as *Mikania micrantha*, show no kin recognition [22]. These studies on kin recognition in clonal plants have focused on intraspecific interactions between individuals of varying relatedness. It therefore remains an open question whether the outcomes of interactions between plants and neighbors that differ in phylogenetic relatedness are identity-dependent. More broadly, clonality can fundamentally alter plant–plant interactions: incorporating clonal traits substantially alters predictions of the Stress-Gradient Hypothesis [23] and considering clonal integration modifies mycorrhizal effects [24]. Together, these findings underscore the need to integrate clonality more explicitly into plant–plant interaction frameworks. Yet, its role remains underappreciated.

Here, we paired the clonal plant *Glechoma grandis* with conspecific, congeneric, or distant heterospecific neighbors and manipulated above- and belowground interaction modes to test whether multi-channel integration facilitates neighbor identity-dependent growth response and whether distinct interaction modes produce a divergent neighbor effect.

## 2. Results

### 2.1. Overall Effects

The growth and allocation of *G. grandis* were significantly affected by leaf contact (Touch), neighbor identity (Neighbor), and their interaction (T × N) (*p* < 0.05), with the Neighbor × shared rooting space interaction (N × C) showing a marginally significant effect (*p* = 0.054). Among all factors, Touch exerted the strongest influence (R^2^ = 0.109, *p* = 0.001) (Table 1).

Touch broadly altered PSL, SSL, SMR, total leaf area, leaf biomass, stolon biomass, and total biomass of *G. grandis*, whereas Connect only affected total ramet number. Neighbor primarily influenced LAR and SLA. Among interactions, C × T was never significant; C × N affected only LAR; T × N strongly modulated LAR, SLA, R/S, RMR, PSL, SMR, and ramet number; and T × C × N exclusively affected PSL (Table 2 and Table 3).

### 2.2. Leaf Contact Effect Across Neighbor Combinations

**Figure 1:**
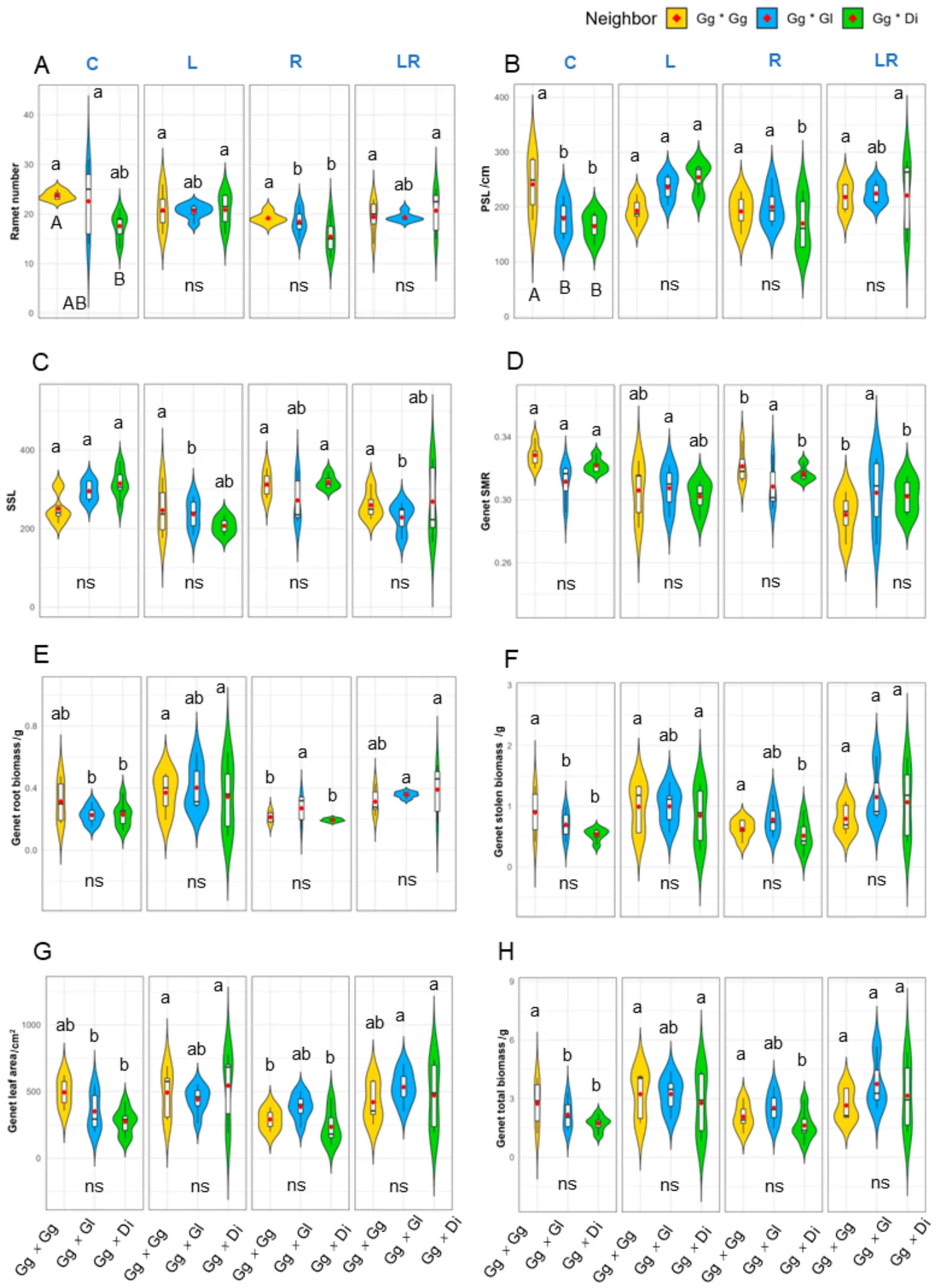
**Effects of treatments on growth parameters of genet.** (**A**) ramet number: the number of ramet; (**B**) PSL: primary stolon length; (**C**) SSL: specific stolon length of the primary stolon; (**D**) SMR: stolon mass ration; (**E**) genet root biomass; (**F**) genet stolon biomass; (**G**) genet leaf area; (**H**) genet total biomass; C: no additional manipulations, root separation with no leaf touch occurring; L: leaf touch manipulation only; R: shared rooting space only; LR: simultaneous leaf touch and shared rooting space. *Gg × Gg*: *Glechoma grandis × Glechoma grandis*, conspecific neighbor combination; *Gg × Gl*: *Glechoma grandis × Glechoma longituba*, congeneric neighbor combination; *Gg × Di*: *Glechoma grandis ×Duchesnea indica*, distant heterospecific neighbor combination. The lowercase letters above the figure indicate a significant difference in the same interaction combination among different treatments, while the capital letter below the figure denotes significant differences among different interaction combinations in the same treatment. The ns means no significant difference. Different letters indicate significant differences, and the same letter or ns means no significant differences at the 0.05 level.

**Figure 2:**
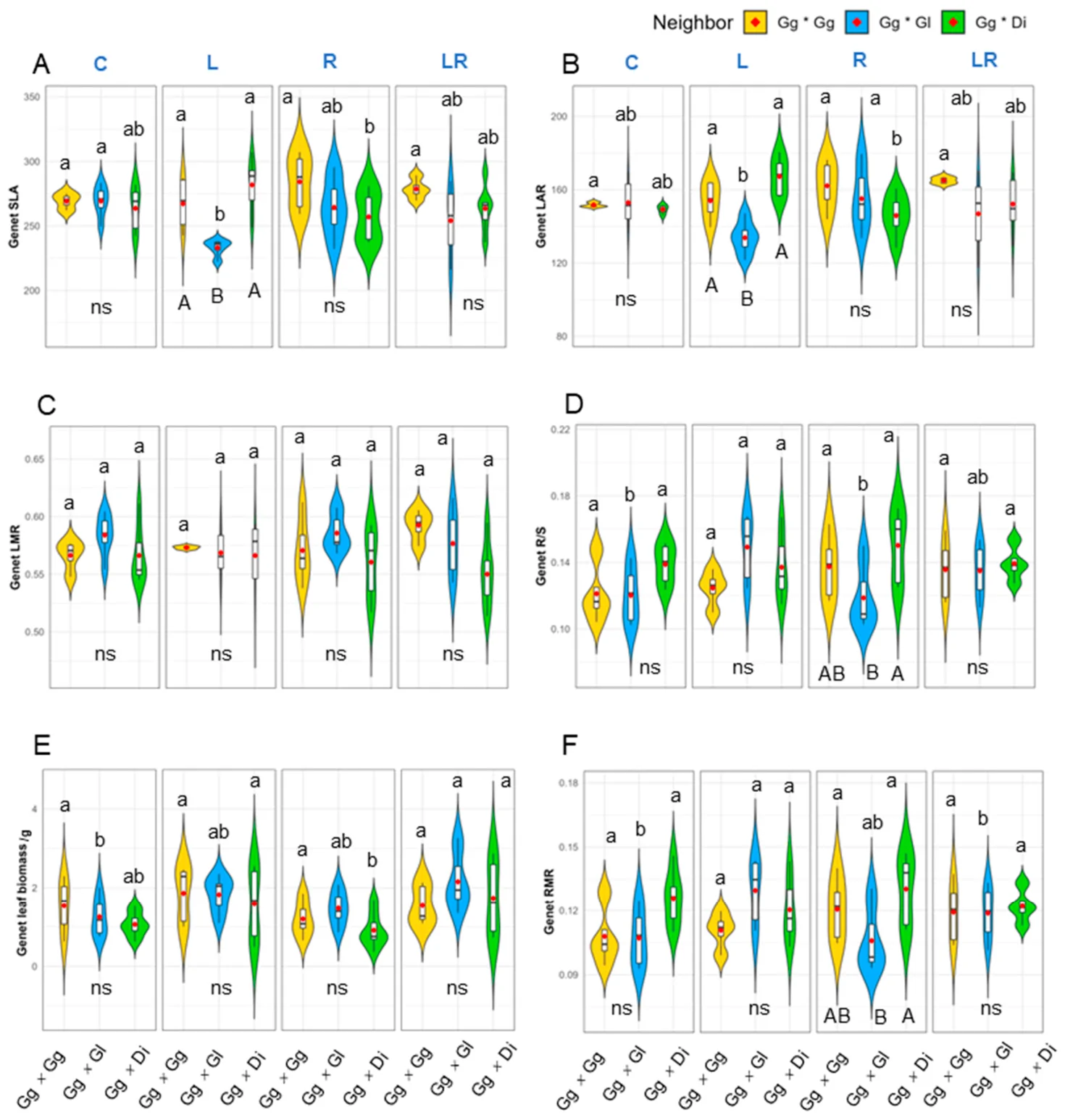
**Effects of treatments on growth parameters of genet (continued).** (**A**) SLA: specific leaf area; (**B**) LAR: leaf area ratio; (**C**) LMR: leaf mass ratio; (**D**) R/S: root biomass/shoot biomass; (**E**) genet leaf biomass; (**F**) RMR: root mass ratio. C: no additional manipulations, root separation with no leaf touch occurring; L: leaf touch manipulation only; R: shared rooting space only; LR: simultaneous leaf touch and shared rooting space. *Gg × Gg*: *Glechoma grandis × Glechoma grandis*, conspecific neighbor combination; *Gg × Gl*: *Glechoma grandis × Glechoma longituba*, congeneric neighbor combination; *Gg × Di*: *Glechoma grandis × Duchesnea indica*, distant heterospecific neighbor combination. The lowercase letters above the figure indicate a significant difference in the same interaction combination among different treatments, while the capital letter below the figure denotes significant differences among different interaction combinations in the same treatment. The ns means no significant difference. Different letters indicate significant differences, and the same letter or ns means no significant differences at the 0.05 level.

We assessed leaf contact effects by comparing the control (C) and leaf contact (L) groups. Leaf contact significantly influenced *G. grandis*, but the responses depended on neighbor identity. In the conspecific combination, no growth parameter of *G. grandis* was significantly altered. In the congeneric combination (*G. grandis* × *G. longituba*), leaf contact reduced SSL and SLA, and increased PSL, total root biomass, RMR, and R/S of *G. grandis*, while its total biomass and ramet number remained unchanged. In the distant heterospecific combination (*G. grandis* × *D. indica*), leaf contact increased PSL, leaf area, root biomass, stolon biomass, and total biomass of *G. grandis* (Figure 1 and Figure 2).

### 2.3. Shared Rooting Space Effects Across Neighbor Combinations

Shared rooting space effects were assessed by comparing the control (C) and shared rooting space groups (R). Shared rooting space significantly influenced *G. grandis* in all combinations. SMR of *G. grandis* decreased in both the conspecific and distant heterospecific combinations, whereas in the congeneric combination, *G. grandis* showed increased PSL and root biomass, but reduced ramet number (Figure 1 and Figure 2).

### 2.4. Combined Leaf Contact and Shared Rooting Space Effects Across Neighbor Combinations

Simultaneous above- and belowground interactions were examined by comparing the control (C) and combined (LR) groups. In the conspecific combination, only SMR of *G. grandis* was reduced. In the congeneric combination, *G. grandis* exhibited decreased SSL but increased total leaf area, root biomass, stolon biomass, leaf biomass, and total biomass. In the distant heterospecific combination, SMR of *G. grandis* decreased, while its PSL, leaf area, root biomass, stolon biomass, and total biomass increased (Figure 1 and Figure 2).

### 2.5. Neighbor Effects Under Different Interaction Modes

We first examined neighbor effects on *G. grandis* in the absence of any interaction (C group). Only PSL and ramet number of *G. grandis* differed among neighbor combinations: plants grown with conspecific neighbors had longer PSL and more ramets than those grown with distant heterospecific neighbors, and longer PSL than those grown with congeneric neighbors; congeneric and distant heterospecific groups did not differ. Under leaf contact alone (L group), *G. grandis* grown with congeneric neighbors exhibited the lowest SLA and LAR, with no significant differences between those grown with conspecific and distant heterospecific neighbors. Under shared rooting space alone (R group), plants grown with distant heterospecific neighbors had higher RMR and R/S than those grown with congeneric neighbors, while those grown with conspecific neighbors did not differ from either. When both leaf contact and shared rooting space were present (LR group), growth of *G. grandis* no longer differed among neighbor combinations (Figure 1 and Figure 2).

## 3. Discussion

### 3.1. Shared Rooting Space Alone Does Not Facilitate Neighbor Identity-Dependent Growth Response

Root exudates can carry specific information about the genetic relatedness, population origin and species identity of neighbors, and trigger different responses at the whole root system level [25]. Plants generally reduce root allocation with kin but increase investment with non-kin [26]. Our findings deviate from this pattern at the interspecific level. Although root communication altered aboveground growth in *G. grandis*, the pattern did not align with the phylogenetic relationship. In the congeneric combination, shared rooting space increased root biomass and primary stolon length of *G. grandis*, while reducing ramet number, indicating enhanced local competitiveness rather than cooperation among congenerics, but at the cost of reducing clonal propagation. Notably, both conspecific and distant heterospecific combinations induced a similar reduction in stolon biomass allocation, indicating that the responses were not tuned to neighbor relatedness. This uniformity contrasts with the divergent strategies observed under leaf contact, where growth adjustments closely tracked neighbor identity. Our findings indicate shared rooting space failed to elicit the expected identity-dependent neighbor responses, suggesting that root-derived effects are overridden when aboveground cues are absent. In our study, the adventitious roots of *G. grandis* and its neighbors did not come into direct contact during the experimental period, which likely limited the exchange of root-derived signals. This insufficient root communication may largely explain why our results differ from those of some previous studies. It also implies that variation in root growth and architecture among species could contribute to the contrasting outcomes observed in plant–plant interactions.

### 3.2. Leaf Contact and Its Combination with Shared Rooting Space Mediate Neighbor Identity-Dependent Growth Response

Our results revealed that the neighbor effect in *G. grandis* depended on the interaction mode: leaf contact between neighbors promoted stronger neighbor identity-dependent response than shared rooting space alone, and the combination of both cues (leaf contact and shared rooting space) produced the strongest effect. Under leaf contact alone, *G. grandis* adjusted its growth depending on neighbor identity—conservative toward conspecifics (no significant changes) but competitive toward heterospecifics, especially distant heterospecifics, by increasing allocation to stolons and roots to enhance horizontal expansion and belowground resource capture. Leaf area allocation further diverged: contact with congenerics reduced leaf area, alleviating light competition, whereas contact with distant heterospecifics increased it, intensifying light competition. When leaf contact and shared rooting space were combined, neighbor identity-dependent response was even more pronounced: toward conspecifics, *G. grandis* adopted a competition-avoidance strategy, reducing stolon biomass to limit spatial spread—a response consistent with altruism; toward heterospecifics, it showed a competitive pattern, increasing leaf area and root, stolon, and leaf biomass to improve fitness. Stolon morphology also responded divergently: congeneric neighbors induced thicker stolons, likely consolidating occupied space, whereas distant heterospecific neighbors promoted longer, thinner stolons that facilitate expansion into new space.

Leaf touch between plants is a form of mechanical stimulus that induces thigmomorphogenesis, typically inhibiting longitudinal elongation and promoting radial expansion [13]. Our results, however, show that thigmomorphogenic responses to neighbor touch were divergent depending on neighbor identity. This implies that leaf contact between neighbors may involve not only physical stimulation but also chemical signaling. It has been proved that mechanical touch triggers hormonal regulation [27], calcium-mediated signaling [6], defense-related gene activation [8], and altered root exudation and microbiota [28]. Compounds derived from these processes likely play a critical role in neighbor identity-dependent response, ultimately driving differential growth adjustments in response to neighbors of varying relatedness. Furthermore, when aboveground leaf contact is combined with belowground root interactions, such multi-channel interaction modes provide more comprehensive neighbor information, thereby facilitating neighbor identity-dependent response.

### 3.3. Neighbor Effects Shift with Interaction Mode and Converge Under Multi-Channel Integration

In the absence of leaf contact and shared rooting space, *G. grandis* distinguished conspecific from heterospecific neighbors but did not differentiate congeneric from distant heterospecifics within heterospecific groups. With conspecifics, plants produced longer stolons and more ramets, a pattern of horizontal expansion and enhanced reproductive competitiveness, with no indication of altruism toward conspecifics. This contrasts with reports in *G. hederacea* and *Fragaria vesca*, where rapid horizontal spread occurs with distant heterospecifics [26], and in *Mimulus guttatus*, where stolon production and reproduction increase specifically with non-kin [29]. These findings suggest that, even without direct contact, *G. grandis* can distinguish a “non-self”, likely via volatile or other airborne cues, that discriminates conspecifics from heterospecifics, but with insufficient resolution to trigger differential responses between congenerics and distant heterospecifics.

When neighbors interacted, the pattern shifted. Conspecific advantages of *G. grandis* in stolon length and ramet number disappeared under all interaction modes, and new differences emerged: leaf contact induced variation in *G. grandis* in leaf area allocation, while shared rooting space generated divergence in root allocation. Notably, regardless of interaction mode, growth did not differ significantly between conspecific and distant heterospecific combinations. When leaf contact and shared rooting space were combined, no significant growth differences in *G. grandis* were detected among any neighbor combinations. Thus, multi-channel interaction between neighbors erased the initial neighbor-driven variation.

This convergence reflects a strategy of genet-level homeostasis in clonal plants: individual ramets execute plastic responses to local signals, while the genet buffers these responses through physiological integration. By acquiring richer neighbor information from multiple channels, *G. grandis* fine-tunes its resource allocation across ramets, offsetting the costs of local competition or cooperation. As a result, whole-genet growth remains stable regardless of neighbor relatedness. Such a buffering mechanism likely enables clonal plants to maintain fitness across heterogeneous social environments, facilitating fine-scale spatial occupancy and long-term coexistence with neighbors of varying relatedness.

## 4. Materials and Methods

### 4.1. Plant Material

We used the stoloniferous clonal herb *Glechoma grandis* (A. Gray) Kupr. as the focal species. As neighbors, we selected *G. longituba* (Nakai) Kupr., a closely related congener (Lamiaceae), and *Duchesnea indica* (Andr.) Focke (Rosaceae), a phylogenetically distant species. All three are perennial herbs that spread vegetatively via creeping stolons which root at nodes, forming networks of connected ramets. *G. longituba* and *D. indica* were collected from Jiwozi in the Qinling Mountains (Shaanxi, China), and *G. grandis* was obtained from the Xi’an Botanical Garden (Shaanxi, China). To minimize genetic variation, all experimental plants were propagated vegetatively from a single genet per species and grown for at least six months in a greenhouse at Northwest University, Xi’an (34.3° N, 108.9° E; 397 m a.s.l.) prior to the experiment, allowing acclimation and reducing carryover effects from the original environments.

### 4.2. Experimental Design

**Figure 3:**
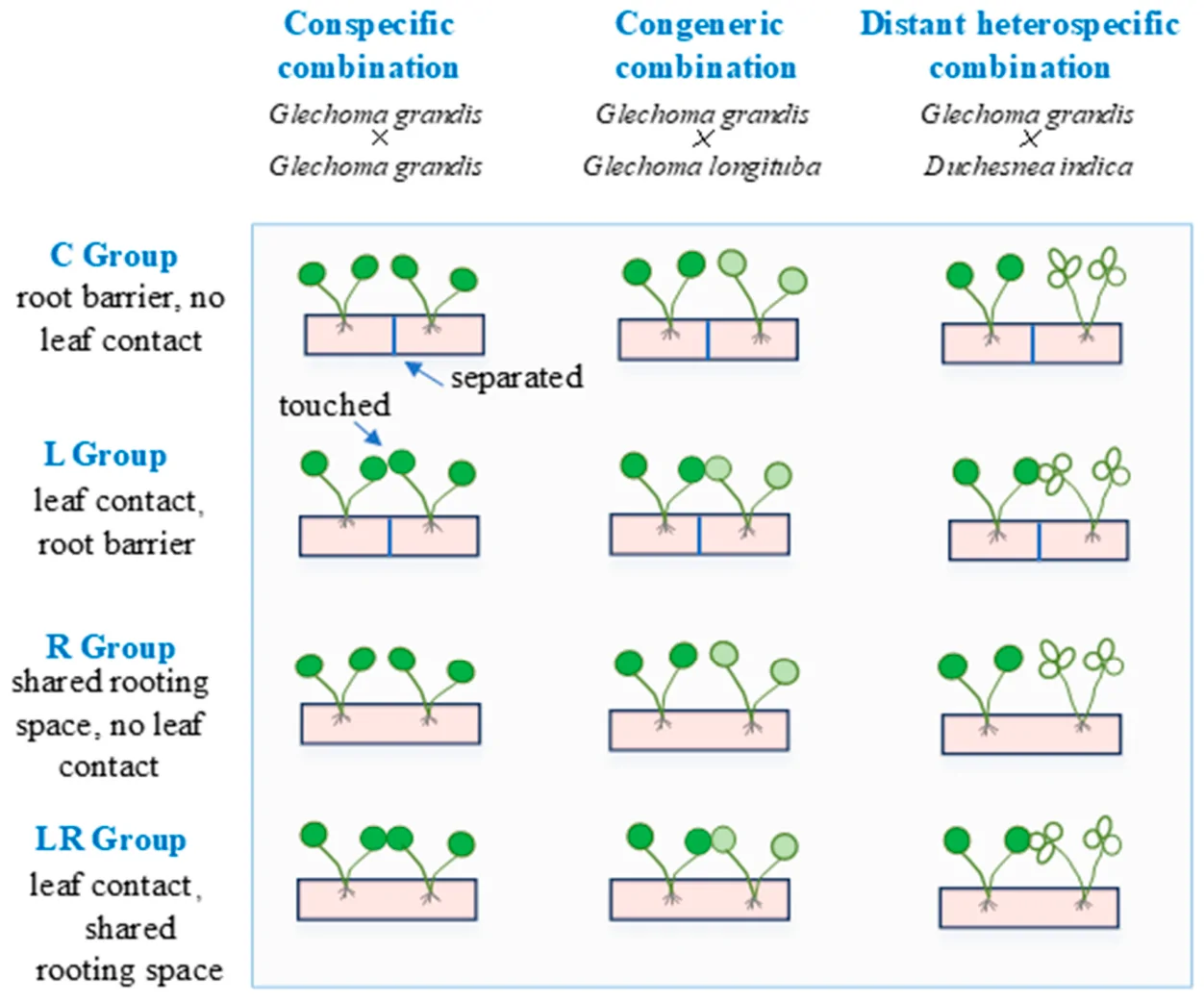
**Schematic representation of the experimental design illustrating the treatment protocols for initial ramet.** C Group: no additional manipulations, root separation with no leaf touch occurring; L Group: leaf touch manipulation only; R Group: shared rooting space only; LR Group: simultaneous leaf touch and shared rooting space. *Glechoma grandis × Glechoma grandis*: conspecific neighbor combination; *Glechoma grandis × Glechoma longituba*: congeneric neighbor combination; *Glechoma grandis ×Duchesnea indica*: distant heterospecific neighbor combination.

The experiment was conducted from 25 July to 8 October 2024, with each experimental unit initiated from a single pair of ramets: a ramet of *G. grandis* was paired with a conspecific (*G. grandis*), a congeneric (*G. longituba*) or a distant heterospecific neighbor (*D. indica*). The two ramets were planted 8 cm apart in containers containing a 4:1:1 (*v*/*v*/*v*) mixture of peat moss, perlite, and vermiculite. We factorially manipulated leaf touch and shared rooting space between neighbors, yielding four treatments: control (C group; root barrier, no leaf contact), leaf contact only (L group; root barrier, manually induced leaf contact), shared rooting space only (R group; shared rooting space, no leaf contact), and combined shared rooting space and leaf contact (LR group; shared rooting space, manually induced leaf contact) (Figure 3). Each of the 3 neighbor combinations × 4 treatments was replicated 10 times (120 pots), arranged randomly in a greenhouse (completely randomized design). The greenhouse was maintained at 24/20 °C (day/night) with a 13/11 h (light/dark) period, photosynthetically active radiation of ~150 μmol·m^−2^·s^−1^, and 40% relative humidity; all plants were watered every three days to prevent water stress. Irrigation was applied directly to the soil surface to avoid unintended leaf contact.

### 4.3. Leaf Touch Treatment

The two initial ramets were planted 8 cm apart in containers, a distance that prevented spontaneous leaf contact during growth. In the leaf touch treatments (L and LR), we followed the protocol of Xing et al.: the paired initial ramets received daily touch stimulation from 08:00 to 18:00 at 2 h intervals, with 10 leaf contacts per session induced by gently bending petioles with a wooden stick [30]. A single operator performed all stimulations to ensure consistency. Only the initial ramets were artificially touched; newly produced offspring ramets were allowed to develop natural contacts but received no additional stimulation, as previous work demonstrated that touch applied exclusively to the initial ramet is sufficient to alter genet-level growth. Petiole bending was minimized to prevent tissue damage, and the touch treatment was maintained throughout the experiment.

### 4.4. Belowground Interaction Manipulation

To manipulate belowground interactions, two contrasting root treatments were established. For root separation treatments (C and L), each initial ramet was planted individually in a plastic container (7.5 × 8.5 × 4.7 cm, length × width × depth), and the two containers were placed adjacently. Because the two ramets were grown in separate containers, their roots were physically isolated and could not connect or interact. For shared rooting space treatments (R and LR), both ramets were co-planted in a single larger container of the same width and depth but twice the length (15 × 8.5 × 4.7 cm). With no physical barrier between the two ramets, their roots could grow freely and interact within the shared soil volume. The soil volume in one large container was twice that in a single small container. Consequently, the belowground treatments differed only in whether roots were physically separated or allowed to connect, while the total soil volume per pair of ramets was kept constant. In the shared rooting space treatments, roots were not physically connected, but could potentially interact through shared soil via resource competition, root exudates, and rhizosphere microbial communities.

### 4.5. Growth and Allocation Measurements

Before planting, the fresh weight of each initial ramet was recorded. At harvest, we recorded the total number of ramets and the primary stolon length (PSL), and measured leaf area and the dry mass of roots, stolons, and leaves of each genet. We calculated the following traits: specific leaf area (SLA; leaf area/leaf biomass), leaf area ratio (LAR; leaf area/total biomass), leaf mass ratio (LMR; leaf mass/total biomass), root–shoot ratio (R/S; root biomass/aboveground biomass), root mass ratio (RMR; root biomass/total biomass), stolon mass ratio (SMR; stolon biomass/total biomass), and specific stolon length (SSL; primary stolon length/primary stolon biomass). As *G. grandis* is a monopodial clonal plant whose primary stolon serves as the main stolon, primary stolon length (PSL) was defined as the length of this main stolon extending directly from the initial ramet, measured from the base of the initial ramet to the stolon apex.

Leaf area was obtained by scanning fresh leaves (Epson Perfection V19, Epson, Beijing, China) and analyzing images with Motic Images Plus 2.0 ML (Motic, Xiamen, China). Dry mass was measured after oven-drying at 80 °C for 72 h to constant mass using an electronic balance (Sartorius BT25S, Sartorius, Beijing, China).

### 4.6. Statistical Analysis

The experiment had three factors: leaf contact (Touch), neighbor species (Neighbor), and shared rooting space (Connect). All models included initial ramet fresh weight as a covariate. To assess the overall multivariate response of growth traits, we first checked multivariate dispersion using PERMDISP (betadisper) based on the same Gower distance matrix, and then used permutational multivariate analysis of variance (PERMANOVA) based on Gower distance, with the three factors and their interactions as predictors and significance assessed via 999 permutations.

For each growth trait, data were first checked for normality and homoscedasticity. When assumptions were met (if necessary, after log transformation), we applied general linear models; otherwise, generalized linear models with a Gamma distribution and a log link function were used. All models included Touch, Neighbor, Connect and their interactions. When a significant interaction was detected, we performed simple-effect analyses to test the effects of Touch and Connect at each level of Neighbor (Gg × Gg, Gg × Gl, Gg × Di). Both simple-effect comparisons and pairwise post hoc comparisons were adjusted using Tukey’s HSD to control the family-wise error rate. All statistical analyses were conducted in R v4.4.0 (R Core Team, 2024), with significance set at α = 0.05.

## 5. Conclusions

Our findings demonstrate that the neighbor effect by *G. grandis* depends on its mode of interaction with neighbors. With only shared rooting space with neighbors, *G. grandis* showed no clear recognition. Leaf contact, however, triggered neighbor identity-dependent growth responses, and when combined with shared rooting space, produced the strongest effect. This indicates that belowground cues alone carry insufficient neighbor information in our experiment system. Notably, the adventitious roots of *G. grandis* and its neighbors did not come into direct physical contact during the experiment, which likely restricted the exchange of root-derived signals. This suggests that the outcome of belowground interactions can depend on root architecture and the extent of root contact, highlighting the need to consider both interaction mode and root traits in studies of plant–plant interaction.

When both aboveground and underground interactions were present, *G. grandis* no longer showed growth differences among neighbor combinations. One possible explanation for this convergence is a strategy of genet-level homeostasis in clonal plants, in which individual ramets execute plastic responses to local signals while the genet buffers these responses through physiological integration. Such a buffering mechanism likely enables clonal plants to sustain fitness in heterogeneous social environments and promotes coexistence with neighbors of varying relatedness. Yet, this hypothesis was not directly tested in our study and should be examined in future work. Taken together, our findings underscore that a full understanding of plant social behavior requires explicit consideration of how interaction modes and root structural characteristics shape information exchange between neighbors. However, we acknowledge that the use of a single genet per species reduces genetic variation within treatments but also limits the generality of our conclusions. The observed responses may reflect the specific genet used and may not be fully representative of the species as a whole. Future studies incorporating multiple genets or populations per species are needed to test the robustness and generality of these findings.

## Figures and Tables

**Table 1:** **Summary of PERMANOVA for main and interaction effects of treatments.** Degrees of freedom followed by R^2^, F and *p* values are given for all the effects analyzed. Significant results (*p* < 0.05) are shown in bold and italicized.

	**Source of Variation**							
	**Initial Biomass**	**Touch** **(T)**	**Neighbor** **(N)**	**Connect** **(C)**	**T × N**	**T × C**	**N × C**	**T × N × C**
***df***	1	1	2	1	2	1	2	2
**R^2^**	0.092	0.109	0.109	0.011	0.048	0.002	0.039	0.012
**F**	9.132	10.904	2.494	1.067	2.405	0.203	1.944	0.602
***p***	***0.001***	***0.001***	***0.016***	0.368	***0.029***	0.924	0.054	0.781

**Table 2:** **Summary of the results of the linear model for main and interaction effects of treatments on growth parameters of genet.** Degrees of freedom (*df*) followed by F or **χ^2^** and *p* values are given for all the effects analyzed. F-tests are applied in general linear models; likelihood ratio tests (reported as a chi-square statistic) in generalized linear models. Significant results (*p* < 0.05) are shown in bold and italicized.

Effect	*df*	LAR		SLA		LMR		R/S		RMR		PSL		SSL		SMR	
		χ^2^	*p*	χ^2^	*p*	χ^2^	*p*	χ^2^	*p*	χ^2^	*p*	χ^2^	*p*	χ^2^	*p*	F	*p*
**Initial biomass**	1	6.255	***0.012***	6.874	***0.009***	7.226	***0.007***	2.3455	0.126	3.039	0.0812	0.001	0.976	0.543	0.461	0.661	0.416
**Connect**	1	0.174	0.676	0.019	0.889	0.127	0.721	2.1605	0.142	1.729	0.188	0.326	0.568	1.923	0.165	2.575	0.109
**Touch**	1	0.421	0.516	0.073	0.787	0.073	0.787	1.1165	0.291	0.8823	0.347	12.428	***0.000***	16.591	***0.000***	22.75	***0.000***
**Neighbor**	2	9.073	***0.011***	12.733	***0.002***	5.181	0.075	4.0975	0.129	5.476	0.065	0.9956	0.608	2.122	0.346	0.856	0.652
**C × T**	1	0.294	0.588	0.111	0.739	0.089	0.765	0.622	0.430	0.364	0.546	0.008	0.929	0.089	0.7645	0.000	0.992
**C × N**	2	6.931	***0.031***	5.129	0.077	3.1001	0.213	5.111	0.078	4.533	0.104	0.952	0.621	5.3183	0.070	1.488	0.475
**T × N**	2	8.935	***0.011***	9.564	***0.008***	3.739	0.155	8.331	***0.016***	8.854	***0.011***	11.408	***0.003***	3.414	0.181	8.162	***0.017***
**C × T × N**	2	0.544	0.583	0.917	0.406	0.632	0.729	0.041	0.960	0.112	0.945	6.761	***0.034***	3.641	0.162	0.060	0.942

LAR: leaf area ratio; SLA: specific leaf area; LMR: leaf mass ratio; R/S: root biomass/shoot biomass; RMR: root mass ratio; LPS: primary stolon length; SSL: specific stolon length of the primary stolon; SMR: stolon mass ratio.

**Table 3:** **Summary of the results of the linear model for main and interaction effects of treatments on growth parameters of genet (continued).** Degrees of freedom (*df*) followed by **χ^2^** and *p* values are given for all the effects analyzed. Significant results (*p* < 0.05) are shown in bold and italicized.

Effect	*df*	Total Leaf Area		Leaf Biomass		Stolon Biomass		Root Biomass		Total Biomass		Ramets Number	
		χ^2^	*p*	χ^2^	*p*	χ^2^	*p*	χ^2^	*p*	χ^2^	*p*	χ^2^	*p*
**Initial biomass**	1	0.614	0.433	0.217	0.642	0.226	0.635	0.449	0.503	0.015	0.902	0.014	0.905
**Connect**	1	2.199	0.138	0.186	0.666	0.014	0.9065	0.746	0.388	0.056	0.813	6.018	***0.014***
**Touch**	1	17.199	***0.000***	15.351	***0.000***	14.214	***0.000***	19.538	***0.000***	16.373	***0.000***	2.242	0.134
**Neighbor**	2	1.935	0.380	4.824	0.089	5.034	0.081	1.327	0.515	5.236	0.073	4.594	0.101
**C × T**	1	0.352	0.553	0.181	0.670	0.784	0.376	0.369	0.544	0.211	0.646	1.874	0.171
**C × N**	2	5.014	0.082	2.919	0.232	3.910	0.142	2.391	0.303	3.118	0.210	0.504	0.777
**T × N**	2	5.491	0.064	1.361	0.506	3.705	0.157	1.957	0.376	2.389	0.303	9.169	***0.010***
**C × T × N**	2	0.549	0.759	0.377	0.828	0.286	0.867	2.517	0.284	0.198	0.906	0.116	0.944

## Data Availability

All data generated or analyzed during this study are included in this published article.

## Abbreviations

PSL	Primary stolon length
SLA	Specific leaf area
LAR	Leaf area ratio
LMR	Leaf mass ratio
R/S	Root–shoot ratio
RMR	Root mass ratio
SMR	Stolon mass ratio
SSL	Specific stolon length
